# Bis(1,3-diethyl­benzimidazolium) tetra­bromidomercurate(II)

**DOI:** 10.1107/S1600536809047461

**Published:** 2009-11-21

**Authors:** Shu-Juan Li, Ai-Hui Chen, Zhan-Ying Zheng, Shu-Wan Liu, Qing-Xiang Liu

**Affiliations:** a Tianjin Key Laboratory of Structure and Performance of Functional Molecules, College of Chemistry and Life Science, Tianjin Normal University, Tianjin 300387, People’s Republic of China; b State Key Laboratory of Element-Organic Chemistry, Nankai University, Tianjin 300071, People’s Republic of China

## Abstract

In the title compound, (C_11_H_15_N_2_)_2_[HgBr_4_], the tetra­coordinated Hg^II^ center of the complex anion adopts a distorted tetra­hedral geometry [Hg—Br = 2.5755 (8)–2.623 (11) Å and Br—Hg—Br = 103.78 (19)–116.4 (3)°]. One of the Br atoms is disordered over two sites [site-occupancy factors = 0.51 (6) and 0.49 (6)]. The N—C—N angles in the cations are 110.7 (6) and 111.4 (7)°. In the crystal packing, a supra­molecular chain is formed *via* both weak inter­molecular C—H⋯Br hydrogen bonds and π–π aromatic ring stacking inter­actions [centroid–centroid separation = 3.803 (1) Å].

## Related literature

For background to the chemistry of imidazolium compounds, see: Bourissou *et al.* (2000 ▶); Garrison & Youngs (2005 ▶); Hunter & Sanders (1990 ▶); Jacobsen *et al.* (2009 ▶); Juan & Lee (1999 ▶). For a related structure, see: Liu *et al.* (2003 ▶).
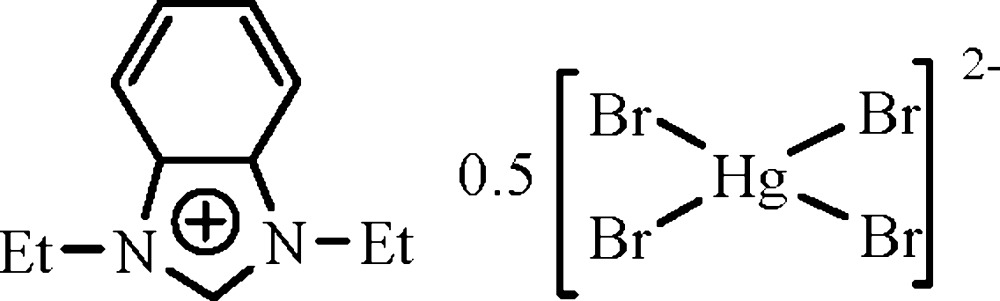


## Experimental

### 

#### Crystal data

(C_11_H_15_N_2_)_2_[HgBr_4_]
*M* *_r_* = 870.73
Triclinic, 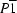

*a* = 8.4334 (15) Å
*b* = 9.9989 (16) Å
*c* = 18.328 (3) Å
α = 85.060 (3)°
β = 81.684 (3)°
γ = 67.250 (2)°
*V* = 1409.5 (4) Å^3^
*Z* = 2
Mo *K*α radiation
μ = 11.15 mm^−1^
*T* = 296 K
0.25 × 0.24 × 0.23 mm

#### Data collection

Bruker MART APEX CCD area-detector diffractometer
Absorption correction: multi-scan (**SADABS**; Sheldrick, 1996 ▶) *T*_min_ = 0.047, *T*_max_ = 0.077
7102 measured reflections
4923 independent reflections
3711 reflections with *I* > 2σ(*I*)
*R*_int_ = 0.019

#### Refinement

*R*[*F* ^2^ > 2σ(*F* ^2^)] = 0.035
*wR*(*F* ^2^) = 0.081
*S* = 1.03
4923 reflections
294 parameters
H-atom parameters constrained
Δρ_max_ = 0.78 e Å^−3^
Δρ_min_ = −0.57 e Å^−3^

### 

Data collection: *SMART* (Bruker, 2003 ▶); cell refinement: *SAINT* (Bruker, 2003 ▶); data reduction: *SAINT*; program(s) used to solve structure: *SHELXS97* (Sheldrick, 2008 ▶); program(s) used to refine structure: *SHELXL97* (Sheldrick, 2008 ▶); molecular graphics: *SHELXTL* (Sheldrick, 2008 ▶); software used to prepare material for publication: *SHELXTL*.

## Supplementary Material

Crystal structure: contains datablocks global, I. DOI: 10.1107/S1600536809047461/zs2017sup1.cif

Structure factors: contains datablocks I. DOI: 10.1107/S1600536809047461/zs2017Isup2.hkl

Additional supplementary materials:  crystallographic information; 3D view; checkCIF report

## Figures and Tables

**Table 1:** Hydrogen-bond geometry (Å, °)

*D*—H⋯*A*	*D*—H	H⋯*A*	*D*⋯*A*	*D*—H⋯*A*
C8—H8*A*⋯Br1′	0.97	2.73	3.69 (2)	175
C8—H8*B*⋯Br4^i^	0.97	2.86	3.755 (8)	153
C28—H28⋯Br1′	0.93	2.84	3.59 (2)	139

Symmetry code: (i) 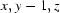.

**Fig. 1.**
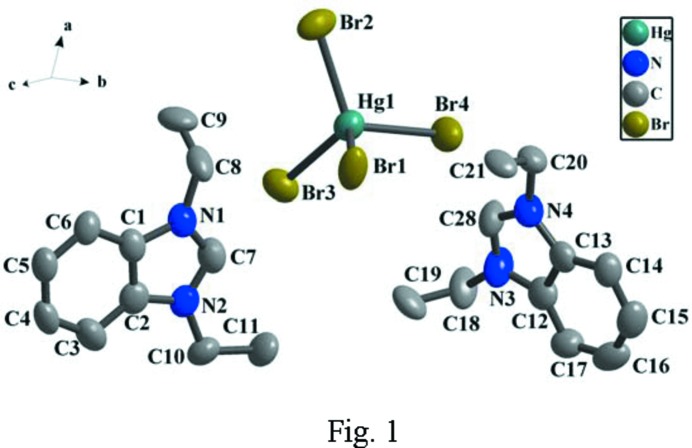
Atom naming scheme for the title compound (I), with displacement ellipsoids drawn at the 30% probability level. All H atoms have been omitted.

**Fig. 2.**
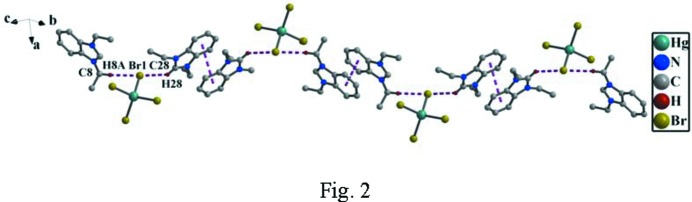
The one-dimensional supramolecular chain of the title compound formed via C—H···Br hydrogen bonds and π-π interactions. The non-interactive H atoms have been omitted.

**Fig. 3.**
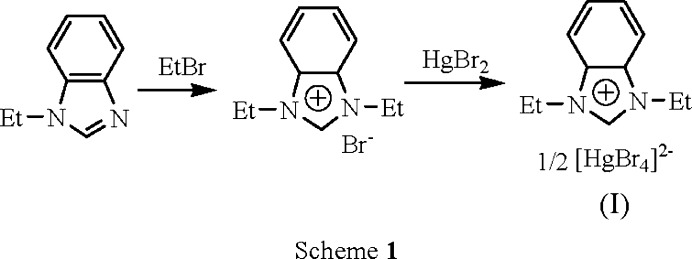
The formation of the title compound.

## supplementary crystallographic information

### Comment

In the last two decades, the complexes of *N*-heterocyclic carbenes (NHCs) have experienced a rapid development and been used in many fields of chemistry (Garrison & Youngs, 2005). Because *N*-heterocyclic carbenes (NHCs) are strong sigma-donors, they have been found to be more effective ligands for organometallic catalysis than other two-electron donor ligands such as phosphines (Bourissou *et al.*, 2000). To date, a number of NHC-metal complexes have been synthesized, and some of these have been used in a broad spectrum of catalytic reactions (Jacobsen *et al.*, 2009). Herein, we report the synthesis and crystal structure of bis(1,3-diethylbenzimidazolium) tetrabromidomercurate(II) 2(C_11_H_15_N_2_)^+^ [HgBr_4_]^2-^ (I) (Fig. 1). In the title compound, the N1—C7, N2—C7 and N3–C28 and N4–C28 bond distances in the two anion are 1.325 (8), 1.301 (8) Å and 1.322 (9), 1.306 (9) Å respectively, and the N1—C7—N2 and N3–C28–N4 bond angles are 110.7 (6) and 111.4 (7)° respectively. These values are similar to those found in 1-(9-anthracenylmethyl)-3-ethylimidazolium iodide (Liu *et al.*, 2003). In the [HgBr_4_]^2-^ complex anion, the tetra-coordinated mercury(II) center adopts a distorted tetrahedral geometry [Hg—Br bond distance range, 2.5755 (8)–2.623 (11) Å; Br–Hg–Br bond angle range, 103.78 (19)–116.4 (3)°]. One of the Br atoms is disordered over two close sites: Br1/Br1' [occupancy factors 0.49 (6), 0.51 (6)]. In the crystal packing of title compound (Fig. 2), a one-dimensional supramolecular chain is formed *via* both weak intermolecular C—H···Br hydrogen bonds (Table 1) (Juan & Lee, 1999), and π-π benzimidazole ring stacking interactions (interplanar centroid-to-centroid separation, 3.803 (1) Å] (Hunter & Sanders, 1990). .

### Experimental

A solution of 1-ethylbenzimidazole (2.00 g, 13.7 mmol) and ethyl bromide (1.64 g, 15.0 mmol) in THF (100 ml) was stirred for three days under reflux, and a white precipitate was formed. The product, 1,3-diethylbenzimidazolium bromide was filtred and washed with THF. Yield: 2.552 g (73%). A suspension of this product (0.200 g, 0.8 mmol) and mercury(II) bromide (0.288 g, 0.8 mmol) in DMSO (5 ml) and acetonitrile (30 ml) was refluxed for 18 h, and a pale yellow solution was formed. Water (30 ml) was added the then extracted with CH_2_Cl_2_ (30 ml). The extract was dried with anhydrous MgSO_4_ and After removing CH_2_Cl_2_, a white powder of the title compound (I) was obtained. Yield, 0.187 g (55%); m.p. 196–198° C; ^1^H NMR (300 MHZ, DMSO-d6): 1.54 (t, J = 4.2, 6H, CH_3_), 3.72 (q, J = 4.2, 4H, CH_2_), 7.35 (m, 2H, PhH), 7.83 (d, J = 6.3, 2H, PhH), 9.22 (s, 1H, 2-bimiH) (bimi = benzimidazole).

### Refinement

All H atoms were initially located in a difference Fourier map. These were then placed in geometrically idealized positions and constrained to ride on their parent atoms, with C*sp*^3^—H = 0.97 Å,C*sp*^2^—H = 0.93 Å and *U*_iso_(H) = 1.2*U*_eq_(C). One of the bromide atoms was found to be disordered over two close sites: Br1/ Br1' [occupancy factors 0.49 (6)/0.51 (6)]. One reflection was considered to be affected by the beamstop.

### Crystal data

**Table:**

(C_11_H_15_N_2_)_2_[HgBr_4_]	*Z* = 2
*M**_r_* = 870.73	*F*(000) = 820
Triclinic, *P*1	*D*_x_ = 2.052 Mg m^−^^3^
Hall symbol: -P 1	Melting point = 469–471 K
*a* = 8.4334 (15) Å	Mo *K*α radiation, λ = 0.71073 Å
*b* = 9.9989 (16) Å	Cell parameters from 3130 reflections
*c* = 18.328 (3) Å	θ = 2.5–25.5°
α = 85.060 (3)°	µ = 11.15 mm^−^^1^
β = 81.684 (3)°	*T* = 296 K
γ = 67.250 (2)°	Block, colourless
*V* = 1409.5 (4) Å^3^	0.25 × 0.24 × 0.23 mm

### Data collection

**Table:**

Bruker SMART APEX CCD area-detector diffractometer	4923 independent reflections
Radiation source: fine-focus sealed tube	3711 reflections with *I* > 2σ(*I*)
graphite	*R*_int_ = 0.019
φ and ω scans	θ_max_ = 25.0°, θ_min_ = 1.1°
Absorption correction: multi-scan (*SADABS*; Sheldrick, 1996)	*h* = −10→7
*T*_min_ = 0.047, *T*_max_ = 0.077	*k* = −11→11
7102 measured reflections	*l* = −20→21

### Refinement

**Table:**

Refinement on *F*^2^	Primary atom site location: structure-invariant direct methods
Least-squares matrix: full	Secondary atom site location: difference Fourier map
*R*[*F*^2^ > 2σ(*F*^2^)] = 0.035	Hydrogen site location: inferred from neighbouring sites
*wR*(*F*^2^) = 0.081	H-atom parameters constrained
*S* = 1.02	*w* = 1/[σ^2^(*F*_o_^2^) + (0.0369*P*)^2^ + 1.1891*P*] where *P* = (*F*_o_^2^ + 2*F*_c_^2^)/3
4923 reflections	(Δ/σ)_max_ = 0.002
294 parameters	Δρ_max_ = 0.78 e Å^−^^3^
0 restraints	Δρ_min_ = −0.57 e Å^−^^3^

### Special details

**Table:**

Geometry. All e.s.d.'s (except the e.s.d. in the dihedral angle between two l.s. planes) are estimated using the full covariance matrix. The cell e.s.d.'s are taken into account individually in the estimation of e.s.d.'s in distances, angles and torsion angles; correlations between e.s.d.'s in cell parameters are only used when they are defined by crystal symmetry. An approximate (isotropic) treatment of cell e.s.d.'s is used for estimating e.s.d.'s involving l.s. planes.
Refinement. Refinement of *F*^2^ against ALL reflections. The weighted *R*-factor *wR* and goodness of fit *S* are based on *F*^2^, conventional *R*-factors *R* are based on *F*, with *F* set to zero for negative *F*^2^. The threshold expression of *F*^2^ > σ(*F*^2^) is used only for calculating *R*-factors(gt) *etc*. and is not relevant to the choice of reflections for refinement. *R*-factors based on *F*^2^ are statistically about twice as large as those based on *F*, and *R*- factors based on ALL data will be even larger.

### Fractional atomic coordinates and isotropic or equivalent isotropic displacement parameters (Å^2^)

**Table:**

	*x*	*y*	*z*	*U*_iso_*/*U*_eq_	Occ. (<1)
Hg1	0.92830 (4)	0.40323 (3)	0.751796 (14)	0.05906 (11)	
N1	0.6000 (7)	0.0822 (6)	0.8460 (3)	0.0596 (14)	
N2	0.3689 (7)	0.2297 (6)	0.9074 (3)	0.0557 (13)	
N3	0.4560 (9)	0.7998 (6)	0.6180 (3)	0.0778 (18)	
N4	0.5979 (8)	0.7150 (6)	0.5126 (3)	0.0657 (15)	
C1	0.5169 (9)	−0.0033 (7)	0.8841 (3)	0.0531 (16)	
C2	0.3671 (9)	0.0921 (7)	0.9230 (3)	0.0557 (16)	
C3	0.2517 (9)	0.0427 (9)	0.9661 (4)	0.0670 (19)	
H3	0.1510	0.1063	0.9917	0.080*	
C4	0.2912 (12)	−0.1027 (10)	0.9697 (4)	0.079 (2)	
H4	0.2162	−0.1396	0.9984	0.095*	
C5	0.4422 (12)	−0.1974 (9)	0.9311 (4)	0.077 (2)	
H5	0.4652	−0.2963	0.9352	0.092*	
C6	0.5578 (10)	−0.1508 (7)	0.8874 (4)	0.0655 (19)	
H6	0.6579	−0.2149	0.8616	0.079*	
C7	0.5054 (10)	0.2196 (7)	0.8613 (4)	0.0616 (18)	
H7	0.5329	0.2980	0.8418	0.074*	
C8	0.7646 (12)	0.0302 (9)	0.7957 (4)	0.088 (3)	
H8A	0.7680	0.1084	0.7612	0.106*	
H8B	0.7669	−0.0485	0.7675	0.106*	
C9	0.9214 (12)	−0.0215 (11)	0.8349 (6)	0.117 (3)	
H9A	0.9124	0.0509	0.8682	0.175*	
H9B	1.0224	−0.0386	0.7996	0.175*	
H9C	0.9305	−0.1100	0.8622	0.175*	
C10	0.2278 (10)	0.3638 (8)	0.9367 (4)	0.077 (2)	
H10A	0.1171	0.3601	0.9309	0.093*	
H10B	0.2326	0.3672	0.9890	0.093*	
C11	0.2388 (12)	0.4979 (8)	0.8993 (5)	0.101 (3)	
H11A	0.3416	0.5084	0.9098	0.151*	
H11B	0.1389	0.5802	0.9168	0.151*	
H11C	0.2432	0.4920	0.8470	0.151*	
C12	0.3604 (10)	0.8877 (7)	0.5645 (4)	0.0619 (18)	
C13	0.4523 (9)	0.8350 (7)	0.4965 (4)	0.0545 (16)	
C14	0.3935 (11)	0.9000 (8)	0.4311 (4)	0.072 (2)	
H14	0.4537	0.8644	0.3858	0.086*	
C15	0.2428 (12)	1.0190 (10)	0.4362 (5)	0.087 (2)	
H15	0.1996	1.0669	0.3932	0.104*	
C16	0.1514 (11)	1.0707 (9)	0.5050 (6)	0.087 (3)	
H16	0.0478	1.1511	0.5063	0.104*	
C17	0.2093 (10)	1.0075 (8)	0.5694 (5)	0.077 (2)	
H17	0.1493	1.0436	0.6147	0.092*	
C18	0.4168 (17)	0.8111 (10)	0.6994 (4)	0.126 (4)	
H18A	0.5189	0.8092	0.7186	0.151*	
H18B	0.3254	0.9048	0.7102	0.151*	
C19	0.3668 (13)	0.7051 (11)	0.7371 (5)	0.116 (3)	
H19A	0.2707	0.7005	0.7165	0.174*	
H19B	0.3332	0.7282	0.7882	0.174*	
H19C	0.4619	0.6129	0.7327	0.174*	
C20	0.7368 (11)	0.6199 (9)	0.4597 (5)	0.094 (3)	
H20A	0.7525	0.6768	0.4156	0.113*	
H20B	0.8445	0.5822	0.4815	0.113*	
C21	0.6995 (15)	0.5009 (11)	0.4393 (6)	0.131 (4)	
H21A	0.6815	0.4452	0.4828	0.197*	
H21B	0.7950	0.4402	0.4066	0.197*	
H21C	0.5968	0.5374	0.4149	0.197*	
C28	0.5942 (12)	0.6992 (8)	0.5842 (4)	0.082 (2)	
H28	0.6782	0.6263	0.6084	0.099*	
Br1	0.7857 (14)	0.3331 (10)	0.6509 (7)	0.068 (2)	0.49 (6)
Br1'	0.745 (4)	0.3411 (16)	0.6681 (16)	0.096 (3)	0.51 (6)
Br2	1.23998 (10)	0.21193 (8)	0.74047 (4)	0.0769 (2)	
Br3	0.77611 (10)	0.41151 (9)	0.88750 (4)	0.0735 (2)	
Br4	0.92020 (11)	0.66240 (8)	0.71463 (5)	0.0789 (2)	

### Atomic displacement parameters (Å^2^)

**Table:**

	*U*^11^	*U*^22^	*U*^33^	*U*^12^	*U*^13^	*U*^23^
Hg1	0.06174 (18)	0.05316 (17)	0.06080 (17)	−0.01868 (13)	−0.01052 (12)	−0.00390 (12)
N1	0.075 (4)	0.053 (3)	0.053 (3)	−0.029 (3)	−0.006 (3)	−0.002 (3)
N2	0.064 (4)	0.053 (3)	0.052 (3)	−0.022 (3)	−0.017 (3)	0.000 (3)
N3	0.113 (5)	0.057 (4)	0.051 (3)	−0.021 (4)	−0.004 (4)	0.000 (3)
N4	0.076 (4)	0.054 (3)	0.059 (4)	−0.022 (3)	0.005 (3)	−0.003 (3)
C1	0.070 (5)	0.059 (4)	0.040 (3)	−0.034 (4)	−0.013 (3)	0.002 (3)
C2	0.065 (4)	0.059 (4)	0.050 (4)	−0.027 (4)	−0.022 (3)	0.001 (3)
C3	0.064 (5)	0.084 (5)	0.065 (4)	−0.043 (4)	−0.008 (4)	0.007 (4)
C4	0.098 (6)	0.098 (6)	0.069 (5)	−0.067 (6)	−0.020 (5)	0.016 (5)
C5	0.108 (7)	0.070 (5)	0.071 (5)	−0.052 (5)	−0.023 (5)	0.003 (4)
C6	0.094 (6)	0.055 (4)	0.054 (4)	−0.031 (4)	−0.019 (4)	−0.003 (3)
C7	0.081 (5)	0.058 (4)	0.055 (4)	−0.037 (4)	−0.011 (4)	0.002 (3)
C8	0.123 (8)	0.065 (5)	0.067 (5)	−0.039 (5)	0.033 (5)	−0.009 (4)
C9	0.072 (6)	0.111 (7)	0.147 (9)	−0.022 (6)	0.035 (6)	−0.037 (7)
C10	0.064 (5)	0.068 (5)	0.091 (5)	−0.012 (4)	−0.020 (4)	−0.002 (4)
C11	0.112 (7)	0.052 (5)	0.135 (8)	−0.027 (5)	−0.015 (6)	−0.004 (5)
C12	0.075 (5)	0.046 (4)	0.068 (5)	−0.028 (4)	−0.001 (4)	−0.007 (3)
C13	0.063 (4)	0.051 (4)	0.057 (4)	−0.032 (3)	−0.003 (3)	0.000 (3)
C14	0.094 (6)	0.074 (5)	0.060 (4)	−0.043 (5)	−0.021 (4)	0.002 (4)
C15	0.102 (7)	0.090 (6)	0.085 (6)	−0.047 (6)	−0.047 (5)	0.016 (5)
C16	0.066 (5)	0.069 (5)	0.128 (8)	−0.022 (4)	−0.023 (5)	−0.021 (5)
C17	0.074 (5)	0.071 (5)	0.081 (6)	−0.023 (4)	−0.002 (4)	−0.016 (4)
C18	0.211 (12)	0.091 (7)	0.059 (5)	−0.045 (8)	0.008 (6)	−0.011 (5)
C19	0.137 (9)	0.141 (9)	0.079 (6)	−0.075 (7)	0.040 (6)	−0.030 (6)
C20	0.093 (6)	0.092 (6)	0.085 (6)	−0.031 (5)	0.019 (5)	−0.013 (5)
C21	0.144 (10)	0.132 (9)	0.118 (8)	−0.063 (8)	0.055 (7)	−0.066 (7)
C28	0.118 (7)	0.054 (5)	0.059 (5)	−0.018 (5)	−0.004 (5)	0.002 (4)
Br1	0.083 (3)	0.054 (3)	0.073 (3)	−0.024 (3)	−0.031 (2)	−0.003 (2)
Br1'	0.135 (8)	0.088 (4)	0.097 (7)	−0.065 (4)	−0.065 (6)	0.026 (3)
Br2	0.0681 (5)	0.0800 (5)	0.0583 (4)	0.0027 (4)	−0.0134 (4)	−0.0127 (4)
Br3	0.0739 (5)	0.0840 (5)	0.0668 (4)	−0.0379 (4)	0.0083 (4)	−0.0146 (4)
Br4	0.0853 (6)	0.0524 (4)	0.0948 (6)	−0.0268 (4)	0.0065 (4)	−0.0060 (4)

### Geometric parameters (Å, °)

**Table:**

Hg1—Br2	2.5755 (8)	C9—H9B	0.9600
Hg1—Br1'	2.594 (11)	C9—H9C	0.9600
Hg1—Br4	2.5994 (9)	C10—C11	1.482 (10)
Hg1—Br3	2.6211 (8)	C10—H10A	0.9700
Hg1—Br1	2.623 (11)	C10—H10B	0.9700
N1—C7	1.325 (8)	C11—H11A	0.9600
N1—C1	1.389 (8)	C11—H11B	0.9600
N1—C8	1.482 (9)	C11—H11C	0.9600
N2—C7	1.301 (8)	C12—C17	1.368 (10)
N2—C2	1.386 (8)	C12—C13	1.396 (9)
N2—C10	1.485 (8)	C13—C14	1.376 (9)
N3—C28	1.322 (9)	C14—C15	1.362 (11)
N3—C12	1.385 (9)	C14—H14	0.9300
N3—C18	1.484 (10)	C15—C16	1.406 (12)
N4—C28	1.306 (9)	C15—H15	0.9300
N4—C13	1.392 (8)	C16—C17	1.353 (11)
N4—C20	1.478 (9)	C16—H16	0.9300
C1—C6	1.377 (9)	C17—H17	0.9300
C1—C2	1.393 (9)	C18—C19	1.384 (12)
C2—C3	1.377 (9)	C18—H18A	0.9700
C3—C4	1.358 (10)	C18—H18B	0.9700
C3—H3	0.9300	C19—H19A	0.9600
C4—C5	1.393 (11)	C19—H19B	0.9600
C4—H4	0.9300	C19—H19C	0.9600
C5—C6	1.367 (10)	C20—C21	1.434 (12)
C5—H5	0.9300	C20—H20A	0.9700
C6—H6	0.9300	C20—H20B	0.9700
C7—H7	0.9300	C21—H21A	0.9600
C8—C9	1.488 (12)	C21—H21B	0.9600
C8—H8A	0.9700	C21—H21C	0.9600
C8—H8B	0.9700	C28—H28	0.9300
C9—H9A	0.9600		
			
Br2—Hg1—Br1'	110.3 (7)	N2—C10—H10A	109.0
Br2—Hg1—Br4	111.94 (3)	C11—C10—H10B	109.0
Br1'—Hg1—Br4	108.4 (3)	N2—C10—H10B	109.0
Br2—Hg1—Br3	111.20 (3)	H10A—C10—H10B	107.8
Br1'—Hg1—Br3	107.5 (8)	C10—C11—H11A	109.5
Br4—Hg1—Br3	107.32 (3)	C10—C11—H11B	109.5
Br2—Hg1—Br1	103.78 (19)	H11A—C11—H11B	109.5
Br4—Hg1—Br1	106.1 (3)	C10—C11—H11C	109.5
Br3—Hg1—Br1	116.4 (3)	H11A—C11—H11C	109.5
C7—N1—C1	108.2 (6)	H11B—C11—H11C	109.5
C7—N1—C8	125.5 (6)	C17—C12—N3	131.7 (7)
C1—N1—C8	126.3 (6)	C17—C12—C13	121.8 (7)
C7—N2—C2	108.9 (6)	N3—C12—C13	106.4 (6)
C7—N2—C10	127.7 (6)	C14—C13—N4	132.6 (7)
C2—N2—C10	123.3 (6)	C14—C13—C12	121.4 (7)
C28—N3—C12	107.9 (6)	N4—C13—C12	106.0 (6)
C28—N3—C18	124.0 (7)	C15—C14—C13	116.7 (7)
C12—N3—C18	128.1 (7)	C15—C14—H14	121.7
C28—N4—C13	108.2 (6)	C13—C14—H14	121.7
C28—N4—C20	124.3 (7)	C14—C15—C16	121.2 (7)
C13—N4—C20	127.5 (6)	C14—C15—H15	119.4
C6—C1—N1	132.2 (7)	C16—C15—H15	119.4
C6—C1—C2	121.9 (6)	C17—C16—C15	122.2 (8)
N1—C1—C2	105.9 (6)	C17—C16—H16	118.9
C3—C2—N2	132.6 (7)	C15—C16—H16	118.9
C3—C2—C1	121.2 (6)	C16—C17—C12	116.6 (7)
N2—C2—C1	106.2 (6)	C16—C17—H17	121.7
C4—C3—C2	117.3 (7)	C12—C17—H17	121.7
C4—C3—H3	121.4	C19—C18—N3	116.4 (8)
C2—C3—H3	121.4	C19—C18—H18A	108.2
C3—C4—C5	121.1 (7)	N3—C18—H18A	108.2
C3—C4—H4	119.4	C19—C18—H18B	108.2
C5—C4—H4	119.4	N3—C18—H18B	108.2
C6—C5—C4	122.7 (7)	H18A—C18—H18B	107.3
C6—C5—H5	118.7	C18—C19—H19A	109.5
C4—C5—H5	118.7	C18—C19—H19B	109.5
C5—C6—C1	115.9 (7)	H19A—C19—H19B	109.5
C5—C6—H6	122.1	C18—C19—H19C	109.5
C1—C6—H6	122.1	H19A—C19—H19C	109.5
N2—C7—N1	110.7 (6)	H19B—C19—H19C	109.5
N2—C7—H7	124.6	C21—C20—N4	112.5 (7)
N1—C7—H7	124.6	C21—C20—H20A	109.1
N1—C8—C9	113.4 (7)	N4—C20—H20A	109.1
N1—C8—H8A	108.9	C21—C20—H20B	109.1
C9—C8—H8A	108.9	N4—C20—H20B	109.1
N1—C8—H8B	108.9	H20A—C20—H20B	107.8
C9—C8—H8B	108.9	C20—C21—H21A	109.5
H8A—C8—H8B	107.7	C20—C21—H21B	109.5
C8—C9—H9A	109.5	H21A—C21—H21B	109.5
C8—C9—H9B	109.5	C20—C21—H21C	109.5
H9A—C9—H9B	109.5	H21A—C21—H21C	109.5
C8—C9—H9C	109.5	H21B—C21—H21C	109.5
H9A—C9—H9C	109.5	N4—C28—N3	111.4 (7)
H9B—C9—H9C	109.5	N4—C28—H28	124.3
C11—C10—N2	113.0 (6)	N3—C28—H28	124.3
C11—C10—H10A	109.0		
			
C7—N1—C1—C6	179.5 (7)	C28—N3—C12—C17	−179.0 (8)
C8—N1—C1—C6	0.5 (11)	C18—N3—C12—C17	1.1 (14)
C7—N1—C1—C2	0.0 (7)	C28—N3—C12—C13	−1.2 (8)
C8—N1—C1—C2	−179.1 (7)	C18—N3—C12—C13	178.8 (8)
C7—N2—C2—C3	−178.2 (7)	C28—N4—C13—C14	179.4 (8)
C10—N2—C2—C3	−1.6 (10)	C20—N4—C13—C14	0.0 (12)
C7—N2—C2—C1	1.6 (7)	C28—N4—C13—C12	−0.9 (8)
C10—N2—C2—C1	178.2 (6)	C20—N4—C13—C12	179.7 (7)
C6—C1—C2—C3	−0.7 (9)	C17—C12—C13—C14	−1.0 (10)
N1—C1—C2—C3	178.9 (6)	N3—C12—C13—C14	−179.0 (6)
C6—C1—C2—N2	179.4 (5)	C17—C12—C13—N4	179.3 (6)
N1—C1—C2—N2	−1.0 (6)	N3—C12—C13—N4	1.3 (7)
N2—C2—C3—C4	−179.5 (6)	N4—C13—C14—C15	−179.6 (7)
C1—C2—C3—C4	0.7 (10)	C12—C13—C14—C15	0.8 (10)
C2—C3—C4—C5	−0.2 (11)	C13—C14—C15—C16	−0.9 (11)
C3—C4—C5—C6	−0.4 (12)	C14—C15—C16—C17	1.3 (13)
C4—C5—C6—C1	0.4 (11)	C15—C16—C17—C12	−1.4 (12)
N1—C1—C6—C5	−179.3 (6)	N3—C12—C17—C16	178.7 (8)
C2—C1—C6—C5	0.2 (9)	C13—C12—C17—C16	1.3 (11)
C2—N2—C7—N1	−1.7 (7)	C28—N3—C18—C19	−71.1 (14)
C10—N2—C7—N1	−178.1 (6)	C12—N3—C18—C19	108.8 (11)
C1—N1—C7—N2	1.1 (7)	C28—N4—C20—C21	91.6 (11)
C8—N1—C7—N2	−179.8 (7)	C13—N4—C20—C21	−89.0 (10)
C7—N1—C8—C9	97.9 (9)	C13—N4—C28—N3	0.2 (9)
C1—N1—C8—C9	−83.2 (9)	C20—N4—C28—N3	179.6 (7)
C7—N2—C10—C11	9.0 (10)	C12—N3—C28—N4	0.7 (10)
C2—N2—C10—C11	−166.8 (6)	C18—N3—C28—N4	−179.4 (8)

### Hydrogen-bond geometry (Å, °)

**Table:**

*D*—H···*A*	*D*—H	H···*A*	*D*···*A*	*D*—H···*A*
C8—H8A···Br1'	0.97	2.73	3.69 (2)	175
C8—H8B···Br4^i^	0.97	2.86	3.755 (8)	153
C28—H28···Br1'	0.93	2.84	3.59 (2)	139

Symmetry codes: (i) *x*, *y*−1, *z*.
